# Dual test gas pulmonary diffusing capacity in patients with idiopathic scoliosis 40 years after diagnosis

**DOI:** 10.1113/EP092251

**Published:** 2025-01-16

**Authors:** Rie S. Thomsen, Milan Mohammad, Lærke C. Ragborg, Casper Dragsted, Søren Ohrt‐Nissen, Martin Gehrchen, Benny Dahl, Ronan M. G. Berg, Jann Mortensen

**Affiliations:** ^1^ Centre for Physical Activity Research Copenhagen University Hospital – Rigshospitalet Copenhagen Denmark; ^2^ Spine Unit, Department of Orthopaedic Surgery Copenhagen University Hospital – Rigshospitalet Copenhagen Denmark; ^3^ Department of Clinical Medicine, Faculty of Health and Medical Sciences University of Copenhagen Copenhagen Denmark; ^4^ Department of Orthopedics and Scoliosis Surgery Texas Children's Hospital and Baylor College of Medicine Houston Texas USA; ^5^ Department of Clinical Physiology and Nuclear Medicine Copenhagen University Hospital – Rigshospitalet Copenhagen Denmark; ^6^ Department of Biomedical Sciences, Faculty of Health and Medical Sciences University of Copenhagen Copenhagen Denmark; ^7^ Neurovascular Research Laboratory, Faculty of Life Sciences and Education University of South Wales Pontypridd UK

**Keywords:** alveolar–capillary membrane diffusing capacity, Cobb angle, lung function, pulmonary capillary blood volume, spine surgery

## Abstract

There is limited knowledge on diffusing capacity in scoliosis patients. It remains to be determined if impaired pulmonary diffusing capacity is mostly influenced by reduced alveolar–capillary membrane diffusing capacity (*D*_M, CO_), reduced pulmonary capillary blood volume (*V*_C_) or both. This study aims to report findings from dual test gas pulmonary diffusing capacity for carbon monoxide and nitric oxide (*D*_L, CO, NO_) with quantification of pulmonary diffusing capacity for carbon monoxide corrected for haemoglobin with a five s breath‐hold (*D*_L, COc, 5s_) and nitric oxide with a five s breath‐hold (*D*_L, NO, 5s_), *D*_M, CO_ and *V*_C_. The study included 57 patients with idiopathic scoliosis seen at our department from 1972 to 1983, all of whom underwent radiological assessment and measurement of *D*_L, CO, NO_ during examination 40 years after diagnosis. One‐way ANOVA was performed for between‐group differences and Pearson's correlation coefficient was used to assess correlations between *D*_L, CO, NO_ metrics and Cobb angle. No significant between‐group differences based on disease severity were detected. Thirty‐nine percent of the patients were presented with either reduced *D*_L, COc, 5s_ or reduced *D*_L, NO, 5s_ represented as *Z*‐scores below −1.65. No significant correlations between Cobb angle and *Z*‐scores for *D*_L, COc, 5s_, *D*_L, NO, 5s_, *D*_M, CO_ and *V*_C_ according to height measurements were found. When using arm span instead, a weak negative correlation between *D*_L, COc, 5s_ and Cobb angle (*r *= −0.29; *P* = 0.04) was detected. In conclusion, approximately 39% of patients with idiopathic scoliosis had either reduced *D*_L, COc, 5s_ or reduced *D*_L, NO, 5s_ 40 years after diagnosis with varying contributions from *V*_C_ or *D*_M, CO_.

## Abstract (highlights)

- **What is the central question of this study?**
  Is pulmonary diffusing capacity for carbon monoxide and nitric oxide affected in patients diagnosed with juvenile or adolescent idiopathic scoliosis 40 years after diagnosis?
- **What is the main finding and its importance?**
  In patients diagnosed with juvenile or adolescent idiopathic scoliosis, about 39% were found to have reduced pulmonary diffusing capacity. No consistent relationship between scoliosis severity and pulmonary diffusing capacity was found.

## 1 INTRODUCTION

It is well‐established that severe cases of idiopathic scoliosis can negatively impact lung function. Previous studies have shown that a larger curve size is associated with an increased reduction in pulmonary volume in untreated patients (Bjure et al., 1970). Additionally, it has been demonstrated that even in patients with mild adolescent idiopathic scoliosis (AIS) there are some reductions in functional exercise capacity (Abdelaal et al., 2018). In contrast, a long‐term follow‐up study of patients with AIS found no correlation between curve size and lung volume (Pehrsson et al., 2001). These somewhat conflicting findings may be caused by the fact that different methods of pulmonary function measurement have been applied in combination with some heterogenicity regarding patient population and treatment.

In idiopathic scoliosis patients with affected pulmonary function, the underlying mechanisms are multifactorial and include spine curvature, thoracic wall deformity, reduced chest wall mobility and consequences from regular scoliosis treatment (Kan et al., 2023). Currently, relatively few studies have investigated the effects of scoliosis on pulmonary function 40 years after diagnosis, and available results are conflicting (Akazawa et al., 2018; Byun et al., 2020; Gitelman et al., 2011; Newton et al., 2005; Ragborg et al., 2024; Ruiz‐Juretschke et al., 2017). From a pathophysiological point of view, the thoracic deformity as a result of scoliosis can potentially damage pulmonary vessels and lead to a decrease in pulmonary volume, but also inhibition of alveolar growth can occur. Collectively, this may lead to a reduced alveolar–capillary membrane surface area and thus compromise the diffusing capacity of the lungs (Siegler & Zorab, 1982; Takahashi et al., 2006; Upadhyay et al., 1995). Accordingly, a recent study from our group has documented that single‐breath pulmonary diffusing capacity for carbon monoxide (*D*_L,CO_) was reduced in 12% of cases (Ragborg et al., 2024). However, it remains to be determined whether this is caused by a reduced alveolar–capillary membrane diffusing capacity (*D*_M,CO_) or a reduced pulmonary capillary blood volume (*V*_C_).

The present study is a secondary analysis from a 40‐year follow‐up study on patients with idiopathic scoliosis including juvenile idiopathic scoliosis (JIS) and AIS (Ragborg et al., 2023). Here, we report findings on dual test gas pulmonary diffusing capacity for carbon monoxide and nitric oxide (*D*_L,CO,NO_), which permits the quantitative assessment of *D*_M,CO_ and *V*_C_. As scoliosis *per se* can impair pulmonary growth of both alveoli and microvasculature, we hypothesised that (1) pulmonary diffusing capacity as assessed by *D*_L,CO,NO_ would be impaired to some degree in idiopathic scoliosis patients 40 years after diagnosis; and (2) pulmonary diffusing capacity expressed as *D*_M,CO_ and *V*_C_ correlates with scoliosis severity.

## 2 METHODS

### 2.1 Approvals

The study was approved by the National Committee on Health and Research Ethics (file no. H‐18000884) and by the Data Protection Agency (2012‐58‐0004) in Denmark. The study conformed to the standards set by the *Declaration of Helsinki*. All participants provided informed written and oral consent.

### 2.2 Study design and setting

This single‐centre cohort study is reported according to the Strengthening the Reporting of Observational Studies in Epidemiology (STROBE) Statement (von Elm et al., 2008). We evaluated patients who were seen at our department at Rigshospitalet in Copenhagen for paediatric spinal deformity from 1972 to 1982.

### 2.3 Participants

One hundred and seventy‐seven patients were registered at our department between April 1972 and April 1982 for a paediatric spinal deformity, and of those, 151 patients were eligible for inclusion at the time 40 years after diagnosis. Full medical records from childhood till follow‐up, pulmonary function tests and radiographic descriptions were reviewed. We excluded all other paediatric deformities than idiopathic scoliosis including JIS and AIS resulting in 129 potential participants from January 2019 to August 2019. Of these, a total of 57 had acceptable combined *D*_L,CO,NO_ measurements performed as a part of their pulmonary function test and these were included in the study.

### 2.4 Variables

#### 2.4.1 Clinical examination

Subjects underwent clinical evaluations including measurement of height, weight, arm span and torsion. They reported on smoking history, previous pulmonary hospitalisations and existing lung diseases like asthma, chronic obstructive pulmonary disease (COPD) or fibrosis. Additionally, breathlessness was assessed using the 1–5 Medical Research Council Dyspnoea Scale.

#### 2.4.2 Assessment of scoliosis

The curvatures were assessed by conventional whole spine, anteroposterior and sagittal X‐rays. Measurements of primary and secondary curves were executed according to the Cobb method (Horng et al., 2019). Information on curve magnitude, location of apex, progression during childhood and scoliosis treatment were collected. Thoracic curves were defined as an apex above Th12 and thoracolumbar/lumbar curves as apex at Th12 or below. The cohort was further divided into three groups depending on disease severity determined from thoracic Cobb angle with mild disease defined as Cobb angle <30°, moderate disease with Cobb angle from 30° to 50° and severe disease with Cobb angle >50°.

#### 2.4.3 Pulmonary function testing

Full standardised pulmonary function tests including dynamic spirometry, whole‐body plethysmography and single‐breath diffusing capacity for CO with a 10 s breath‐hold (*D*_L, CO,10s_) (Jaeger MasterScreen PFT pro, CareFusion, Höchberg, Germany) were conducted in accordance with predetermined guidelines and acceptability and repeatability criteria (Bhakta et al., 2023; Graham et al., 2019). Values of forced expiratory volume in 1 s (FEV_1_), forced vital capacity (FVC), FEV_1_/FVC ratio, total lung capacity (TLC) and residual volume (RV) were conducted as raw values and percentage of predicted in accordance with established reference equations (Quanjer et al., 1993). Height (rounded to the nearest mm), arm span (rounded to the nearest mm), weight (rounded to the nearest 100 g) and Hb (rounded to the nearest 0.1 mmol/L) were collected for each patient. Hb was collected and analysed with the HemoCue Hb 201+ device (HemoCue, Brønshøj, Denmark).

#### 2.4.4 *D*_L,CO,NO_ measurements

*D*_L, CO, NO_ measurements were conducted via a single‐breath manoeuvre in accordance with established quality criteria for clinical use (Munkholm et al., 2018). In short, patients were placed in a sitting position and equipped with a nose clip. The patient commenced normal tidal breathing into a mouthpiece (SpiroBac, Henrotech, Aartselaar, Belgium) connected to a pneumotach (Jaeger MasterScreen PFT pro; equipped dead space, 56 mL; resistance to flow at 12 L s^−1^, 0.9 cmH_2_O). After a few tidal breaths the patient was instructed to perform a maximal expiration to RV followed by a rapid maximal inspiration, at which a valve opened and inhalation of standardised gas fractions (0.28% CO, 9.3% He, 20.9% O_2_ and 69.52 N_2_ mixed with 800 ppm NO/N_2_) was obtained via an inspiratory bag (CiTicel, City Technology, Nuremberg, Germany). At maximal inspiration, a breath‐hold time of 5 s was obtained before maximal expiration. After a 4‐min wash‐out period, the single‐breath manoeuvre was repeated until two manoeuvres fulfilled the repeatability criteria, which included the difference in diffusing capacity for NO with a five s breath‐hold (*D*_L, NO, 5s_) of less than 5.8 mmol min^−1^ kPa^−1^ and difference in diffusing capacity for CO with a five s breath‐hold (*D*_L, CO, 5s_) of less than 1.0 mmol min^−1^ kPa^−1^ (Zavorsky et al., 2017).

*D*_L,CO_ is mechanically equivalent to the conductance of CO across the alveolar–capillary membrane, through plasma and the red blood interior to Hb. It depends on both the conductance of *D*_M,CO_ and the so‐called specific conductance of pulmonary capillary blood to CO (θ_CO_). The latter depends on both the conductance of CO in blood *per se* and on the reaction rate of CO with Hb. Given that the reciprocal of conductance is resistance, the total resistance to transfer of a test gas depends on the following resistances in series (Roughton & Foster, 1957):

$$\frac{1}{D_{L,\mathrm{CO},5s}}=\frac{1}{D_{M,\mathrm{CO}}}+\frac{1}{\theta_{\mathrm{CO}}\cdot V_{C}}$$

These components may be distinguished by concurrently measuring diffusing capacity to CO and NO, because these have different θ‐values and their respective diffusing capacity thus depends differently on *V*_C_ (Zavorsky et al., 2017). Upon simultaneous measurement of *D*_L, CO, 5s_ and *D*_L, NO, 5s_, *D*_M, CO_ and *V*_C_ can be derived by the following equations (Munkholm et al., 2018):

$$D_{M,\mathrm{CO}}=\frac{\frac{1}{\alpha }-\frac{1}{k}}{\frac{1}{D_{L,\mathrm{NO},5s}}-\frac{1}{k\times D_{L,\mathrm{CO},5s}}}$$

$$V_{C}=\frac{\left(\frac{1}{\theta_{\mathrm{CO}}}\right)\times \left(1-\frac{\alpha }{k}\right)}{\frac{1}{D_{L,\mathrm{CO},5s}}-\frac{\alpha }{D_{L,\mathrm{NO},5s}}}$$

where α was related to the diffusivity ratio $\left(\frac{D_{M,\mathrm{NO}}}{D_{M,\mathrm{CO}}}\right)$, which represented the ratio of physical solubilities of NO and CO in tissue. *k* was the ratio between the specific conductance of the two test gases ($\frac{\theta_{\mathrm{NO}}}{\theta_{\mathrm{CO}}}$). The calculation of *D*_M, CO_ and *V*_C_ from D_L, CO, 5s_ and *D*_L, NO, 5s_ depends on several empirical constants as recommended by the European Respiratory Society (ERS) task force (Borland & Hughes, 2019). α is assumed to be 1.97, while *k* was based on the finite $\theta_{\mathrm{NO}}$ set to 1.51 mL blood min^−1^ kPa^−1^ mmol NO^−1^ (4.5 mL blood min^−1^ mmHg^−1^ mL NO^−1^), and $\theta_{\mathrm{CO}}$ calculated using empirical constants measured at pH 7.4 as $\theta_{\mathrm{CO}}=\frac{1}{3.88\;\left(\text{mmol CO}\min\text{kPa ml bloo}d^{-1}\right)+0.092\left(\text{mmol C}O\min\mathrm{mL}\;\mathrm{bloo}d^{-1}\right)\times P_{c,O_{2}}\left(\mathrm{kPa}\right)}\;\times \frac{\left[\mathrm{Hb}](\mathrm{mM}\right)}{9.06\;(\mathrm{mM})}$, where $P_{c,O_{2}}\left(\mathrm{kPa}\right),$ the capillary oxygen tension, is assumed to be 13.33 and [Hb] is the Hb concentration in capillary blood (mM). Mean values of pulmonary diffusing capacity for carbon monoxide corrected for haemoglobin with a five s breath‐hold (*D*_L, COc, 5s_), *D*_L, NO, 5s_, *D*_M, CO_ and *V*_C_ were reported both as raw values and as percentage of predicted using established reference equations (Munkholm et al., 2018). Furthermore, corresponding *Z*‐scores, as proposed by the American Thoracic Society (ATS) and ERS were calculated from the following equation (Stanojevic et al., 2022):

$$\begin{array}{ccc} & & Z\;\mathrm{score}\\ & & \;=\;\frac{\mathrm{Measured}\;\mathrm{pulmonary}\;\mathrm{variable}-\mathrm{predicted}\;\mathrm{pulmonary}\;\mathrm{variable}}{\mathrm{Residual}\;\mathrm{standard}\;\mathrm{deviation}\;\left(\mathrm{RSD}\right)} \end{array}$$

with the measured pulmonary variable being the mean of the pulmonary variable measured from two acceptable and repeatable manoeuvres and predicted pulmonary variable and RSD determined using established reference equations taking age, sex and height/arm span into consideration (Munkholm et al., 2018). The use of height and arm span, respectively, would be expected to cause differences in *Z*‐scores, as disease severity causes discrepancy between height and arm span. Interpretation of *Z*‐scores was as proposed by ATS and ERS with lower limit of normal and upper limit of normal defined as the 5th and 95th percentile. A *Z*‐score between −1.65 and −2.5 was considered a mild reduction, −2.51 to −4.0 as a moderate reduction and below −4.1 as a severe reduction (Stanojevic et al., 2022).

#### 2.4.5 Primary endpoint

*D*_M,CO_ and *V*_C_ *Z*‐scores are co‐primary outcomes.

### 2.5 Data sources

Pulmonary function data were extracted from a local database (DatGen, version 0.6b) at the Department of Clinical Physiology and Nuclear Medicine at Rigshospitalet in Copenhagen connected to the Department's lung function system. The X‐rays were extracted from the Department's radiology information and picture archiving and communication system (RIS/PACS, Agfa, Mortsel, Belgium) and analysed using the validated imaging software KEOPS (SMAIO, Saint‐Priest, France) (Maillot et al., 2015). Patient demographics, clinical data and information on spine‐related treatment during adulthood were extracted from electronic health records (Epic, Verona, WI, USA).

### 2.6 Potential sources of bias

The risk of misclassification of idiopathic scoliosis in the included patients is considered low as the diagnosis was confirmed at a specialised centre. However, as the study is being conducted at a single centre and primarily on Caucasians, the results may not be generalisable to all patients. The study did not conduct a formal a priori statistical power calculation, and the sample size was based on available data, potentially leading to a type 2 error. Moreover, potential confounding variables such as comorbidities and medication usage that could influence pulmonary function independently of scoliosis severity were not fully accounted for.

### 2.7 Sample size

No formal a priori statistical power calculation was conducted before the study, and the sample size was based on available data during the defined study period.

### 2.8 Statistics

The statistical analysis was performed in R statistical software version 4.3.0 (R Foundation for Statistical Computing, Vienna, Austria) within RStudio (version 2023.09.0). Variables were reported as mean (standard deviation (SD)) if normally distributed data and median [interquartile range (IQR)] if non‐parametric data. To assess the difference in pulmonary function parameters across disease severity, a one‐way ANOVA was performed for normally distributed data and a Kruskal–Wallis test was conducted for non‐parametric data. Additionally, an ANOVA was conducted to test the relationship between smoking, treatment and age of diagnosis on reduced diffusing capacity metrics expressed as *Z*‐scores. Assessment of the correlation between pulmonary function metrics and disease severity was executed by calculation of the Pearson correlation coefficient. For assessment of difference in height and arm span, an unpaired Student's *t*‐test was conducted for normally distributed data and a Mann–Whitney *U*‐test were conducted for non‐parametric data. Scatter plots were employed to elucidate the relationship between *D*_L,COc,5s_, *D*_L,NO,5s_, *D*_M,CO_ and *V*_C_ and Cobb angle. A *P*‐value ≤ 0.05 was considered statistically significant.

## 3 RESULTS

### 3.1 Baseline characteristics

Patient characteristics are provided in Table 1. A total of 57 patients (one male) with a mean age of 54.9 (52.3–55.3) years and time from diagnosis to measurement of 40.2 (6.2) years were included in the study. Twenty‐three patients (40.4%) had a history of former or current smoking. The mean Cobb angle for thoracic curves were 20 (7) degrees for mild scoliosis disease severity, 40 (6) degrees for moderate scoliosis disease severity and 65 (10) degrees for severe disease. Overall, no significant differences between groups in percentage of predicted for standardised pulmonary function testing were found (Table 1). As pulmonary function measurements for dynamic spirometry, body plethysmography and *D*_L,CO,10s_ have been published elsewhere (Ragborg et al., 2024), these parameters will not be elaborated further.

**TABLE 1:** Baseline characteristics.

	Total (*n* = 57)	Mild (*n* = 13)	Moderate (*n* = 22)	Severe (*n* = 22)	*P*
Sex (F/M)	56/1	13/0	21/1	22/0	**–**
Previous or current smoker	23	4	10	9	0.62
Age (years)	54.9 [52.3, 55.3]	54.9 [52.3, 55.1]	54.1 (2.2)	54.7 (2.1)	0.45
Height (cm)	163.4 (6.4)	167.5 (7.9)	163.7 (5.5)	160.7 (4.9)	0.01
Arm span (cm)	166.5 (7.1) *n* = 51	167.5 (8.2) *n* = 13	166.4 (6.5) *n* = 20	166.1 (7.3) *n* = 18	0.88
Weight (kg)	67.1 [62.5, 77.2]	70.2 (13.7)	65.3 [60.5, 78.0]	69.3 (10.9)	0.96
BMI (kg/m^2^)	25.2 [23.5, 28.5]	24.0 [22.3, 25.4]	26.3 (4.9)	26.8 (3.9)	0.45
Time from diagnosis to measurement (years)	40.2 (6.2)	39.4 (2.8)	39.8 (2.7)	40.9 (3.1)	0.35
Cobb angle, thoracic curve (°)	45 (20)	20 (7)	40 (6)	65 (10)	<0.001
Global kyphosis (°)	50.2 (10.1)	49.8 (10.1)	50.4 (9.6)	50.1 (11.0)	0.99
FEV_1_ (L)	2.53 (0.48)	2.81 (0.55)	2.48 (0.32)	2.42 (0.53)	0.06
FEV_1_ (% of predicted)	102.1 (21.5)	110.1 (25.1)	98.5 (17.3)	101.0(22.8)	0.30
FVC (L)	3.27 (0.58)	3.50 (0.67)	3.27 (0.44)	3.14 (0.63)	0.21
FVC (% of predicted)	112.0 (21.9)	116.8 (25.7)	110.3 (20.1)	109.8 (22.3)	0.68
FEV_1_/FVC ratio	0.80 [0.70, 0.80]	0.80 [0.80, 0.80]	0.76 (0.10)	0.77 (0.10)	0.08
FEV_1_/FVC ratio (% of predicted)	91.2 (6.6)	94.7 [93.4, 97.4]	89.6 (6.2)	91.0 (7.0)	0.10
TLC (L)	5.17 (0.73)	5.44 (0.71)	5.20 (0.74)	4.97 (0.71)	0.19
TLC (% of predicted)	103.7 (16.0)	106.8 (17.8)	113.4 (16.8)	101.3 (15.3)	0.09
RV (L)	1.90 (0.38)	1.92 (0.34)	1.94 (0.43)	1.84 (0.34)	0.69
RV (% of predicted)	104.0 (20.5)	104.0 (20.9)	105.9 (23.1)	101.9 (18.2)	0.82

*Note*: Values are presented as mean (SD) or median [IQR]. Abbreviations: F/M, female/male; BMI, body mass index; FEV_1_, forced expiratory volume in 1 s; FVC, forced vital capacity; TLC, total lung capacity; RV, residual volume.

### 3.2 D_L,CO,NO_

**FIGURE 1:**
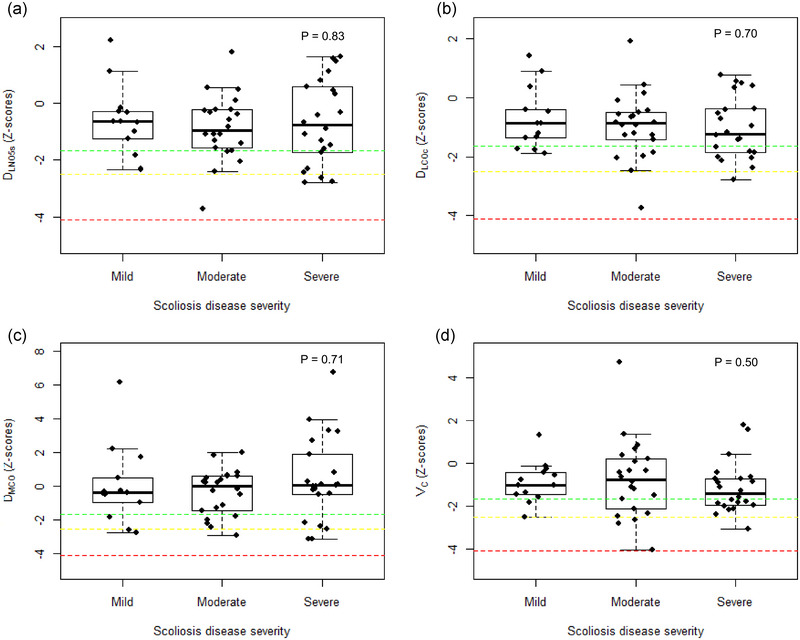
Dual test gas pulmonary diffusing capacity *Z*‐scores based on height according to scoliosis disease severity. Boxplots show median (horizontal line) and percentiles with boxes extending from the 25th percentile to the 75th percentile for each group. The green line indicates mild reduction, the yellow line indicates moderate reduction and the red line indicates severe reduction in the given diffusing capacity metric. *D*_L,COc,5s_, pulmonary diffusing capacity for carbon monoxide corrected for haemoglobin measured with 5 s breath‐hold; *D*_L,NO,5s_, pulmonary diffusing capacity for nitric oxide with a 5 s breath‐hold; *D*_M,CO_, membrane diffusing capacity; *V*_C_, pulmonary capillary blood volume.

**FIGURE B1:**
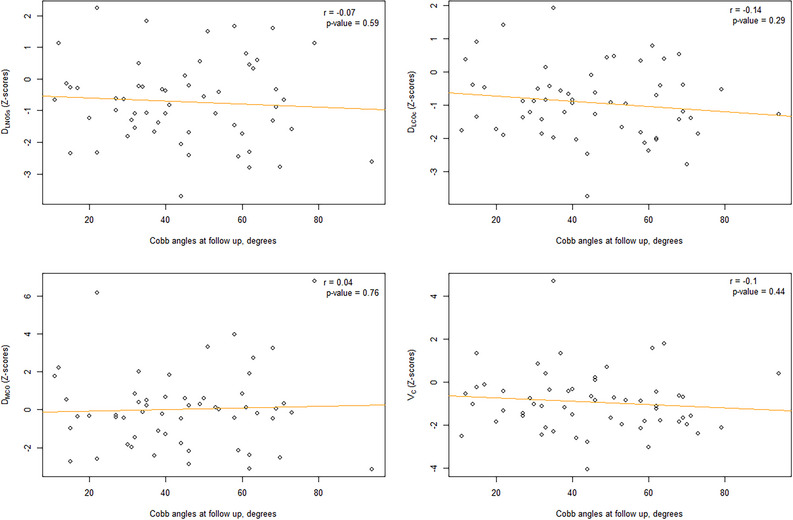
Dual test gas pulmonary diffusing capacity *Z*‐scores based on height versus cobb angle. Abbreviations*: D*_L,COc,5s_, pulmonary diffusing capacity for carbon monoxide corrected for haemoglobin measured with 5 s breath‐hold; *D*_L,NO,5s_, pulmonary diffusing capacity for nitric oxide with 5 s breath‐hold; *D*_M,CO_, membrane diffusing capacity; *V*_C_, pulmonary capillary blood volume.

No overall significant impairment or between‐group differences of any *D*_L,CO,NO_ metrics were found (Table 2, and Appendix A, Table A1). According to calculated *Z*‐scores (*Z*‐scores below −1.65), 39% of the total patients had a reduction in either *D*_L,COc,5s_ or *D*_L,NO,5s_, 28% had a reduction in *D*_L,COc,5s_, 25% had a reduction in *D*_L,NO,5s_, while 23% had a reduction in *D*_M,CO_ and 33% had a reduction in *V*_C_ (Figure 1). In those patients with reduced *D*_L,COc,5s_, the mean *Z*‐score for *D*_M,CO_ was −0.78 and for *V*_C_, the mean *Z*‐score was −2.04. Moreover, in patients with reduced *D*_L,COc,5s_, 19% had reduced *D*_M,CO_, 56% had reduced *V*_C_ and 19% had both reduced *D*_M,CO_ and *V*_C_. No significant correlation was observed between Cobb angle and *Z*‐scores for *D*_L,COc,5s_ when calculated upon height measurements (*r* = −0.14; *P* = 0.29), *D*_L,NO,5s_ (*r* = −0.07; *P* = 0.59), *D*_M,CO_ (*r *= 0.04; *P* = 0.76) and *V*_C_ (*r* = −0.1; *P* = 0.44) (Appendix B, Figure B1). Of note, 38%, 50%, 62% and 47% of the participants with reduced *D*_L,COc,5s_, *D*_L,NO,5s,_ *D*_M,CO_ and *V*_C_, respectively, were either former or current smokers, but the proportion of former or current smoking status did not differ between those with normal versus reduced (Appendix C, Table C1). Additionally, no effect of either age of diagnosis or treatment on the pulmonary diffusing capacity metrics were found (Appendix C, Table C1).

**TABLE 2:** Dual test gas pulmonary diffusing capacity metrics according to scoliosis disease severity.

	Total (*n* = 57)	Mild (*n* = 13)	Moderate (*n* = 22)	Severe (*n* = 22)	*P*
*D*_L,COc,5s_ (mmol/min/kPa)	6.80 (1.14)	7.30 (1.26)	6.76 (1.05)	6.55 (1.10)	0.17
*D*_L,COc,5s_ (% of predicted)	85.24 (12.10)	88.03 (11.30)	86.80 (11.66)	81.80 [75.30, 86.45]	0.63
*D*_L,NO,5s_ (mmol/min/kPa)	30.99 (5.49)	32.74 (5.73)	30.51 (4.54)	30.55 (6.26)	0.52
*D*_L,NO,5s_ (% of predicted)	91.56 (15.27)	93.12 (14.19)	90.37 (13.34)	91.84 (18.04)	0.88
*D*_M,CO_ (mmol/min/kPa)	27.02 [22.22, 30.41]	28.15 (8.82)	25.25 (5.22)	28.46 (9.13)	0.54
*D*_M,CO_ (% of predicted)	98.18 [82.56, 107.7]	100.64 (29.82)	95.17 (17.62)	104.58 (34.53)	0.54
*V*_C_ (mL)	51.29 (43.11–56.64)	53.42 [51.29, 55.62]	50.61 [47.59, 56.92]	48.38 (10.98)	0.10
*V*_C_ (% of predicted)	85.71 [72.34, 94.69]	87.37 (12.81)	89.56 (26.84)	82.63 (17.75)	0.40
*K*_COc_ (mmol/min/kPa/L)	1.53 (0.21)	1.56 (0.43)	1.51 (0.23)	1.47 (0.29)	0.28
*K*_COc_ (% predicted)	98.23 (14.21)	106.19 (26.81)	100.64 (20.10)	94.66 (19.07)	0.30
*K*_NO_ (mmol/min/kPa/L)	7.02 (1.42)	7.20 (1.73)	7.03 (1.32)	6.97 (1.62)	0.66
*K*_NO_ (% of predicted)	102.58 (21.60)	105.89 (25.67)	102.90 (20.00)	99.71 (23.84)	0.58
*V*_A,5s_ (L)	4.48 (0.61)	4.52 (0.66)	4.41 (0.62)	4.52 (0.60)	0.81
*V*_A,5s_ (% of predicted)	90.93 (13.13)	88.62 (14.17)	89.29 (12.18)	93.93 (13.47)	0.40

*Note*: Values are presented as mean (SD) or median [IQR]. Abbreviations: *D*_L,COc,5s_, pulmonary diffusing capacity for carbon monoxide corrected for haemoglobin measured with 5 s breath‐hold; *D*_L,NO,5s_, pulmonary diffusing capacity for nitric oxide measured with 5 s breath‐hold; *D*_M,CO_, membrane diffusing capacity; *V*_C_, pulmonary capillary blood volume; *K*_COc,_ rate of change of CO from alveolar gas corrected for haemoglobin; *K*_NO_, rate of change of NO from alveolar gas; *V*_A,5s_, alveolar volume at 5 s breath‐hold.

### 3.3 Height versus arm span

**FIGURE 2:**
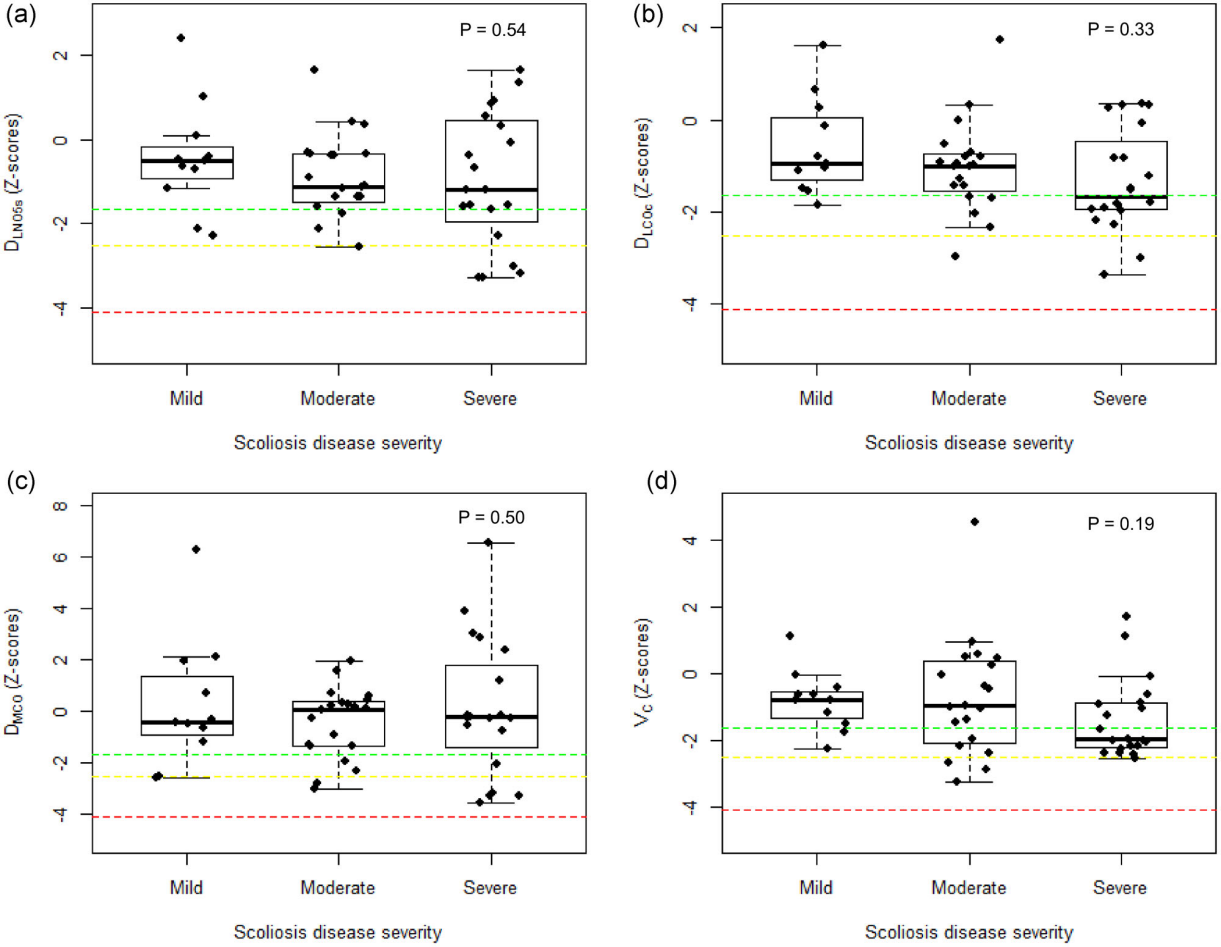
Dual test gas pulmonary diffusing capacity *Z*‐scores based on arm span according to scoliosis disease severity. Boxplots show median (horizontal line) and percentiles with boxes extending from the 25th percentile to the 75th percentile for each group. The green line indicates mild reduction, the yellow line indicates moderate reduction and the red line indicates severe reduction in the given diffusing capacity metric. *D*_L,COc,5s_, pulmonary diffusing capacity for carbon monoxide corrected for haemoglobin measured with 5 s breath‐hold; *D*_L,NO,5s_, pulmonary diffusing capacity for nitric oxide with 5 s breath‐hold; *D*_M,CO_, membrane diffusing capacity; *V*_C_, pulmonary capillary blood volume.

**FIGURE D1:**
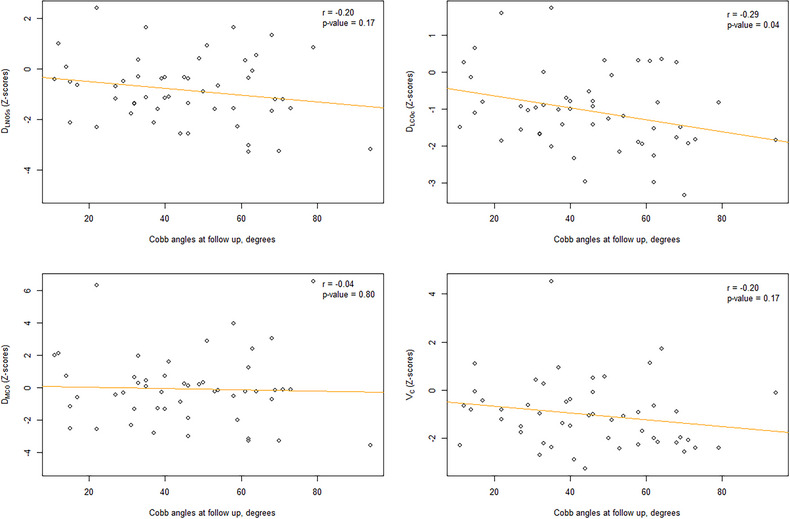
Dual test gas pulmonary diffusing capacity *Z*‐scores based on arm span versus Cobb angle. Abbreviations: *D*_L,COc,5s_, pulmonary diffusing capacity for carbon monoxide corrected for haemoglobin measured with 5 s breath‐hold; *D*_L,NO,5s_, pulmonary diffusing capacity for nitric oxide with 5 s breath‐hold; *D*_M,CO_, membrane diffusing capacity; *V*_C_, pulmonary capillary blood volume.

Measurement of arm span was available for 51 patients. The mean arm span was 2.99 (95% CI: 5.66, 0.31) cm larger than height (*P* = 0.03). When using arm span data instead of height data to calculate *Z*‐scores, *D*_L,COc,5s_, *D*_L,NO,5s_, *D*_M,CO_ and *V*_C_ provided largely similar values with no significant difference between them (Table 3). Based on this, 39% of the patients had a reduction in either *D*_L,COc,5s_ or *D*_L,NO,5s_, 32% of the patients had a reduction in *D*_L,COc,5s_, 22% had a reduction in *D*_L,NO,5s_, 22% had a reduction in *D*_M,CO_ and 39% had a reduction in *V*_C_ (Figure 2). In those patients with reduced *D*_L,COc,5s_ the mean *Z*‐score for *D*_M,CO_ was −1.21 and for *V*_C_, the mean *Z*‐score was −1.81. Scatterplots for association between *Z*‐scores with arm span data and Cobb angle are presented in Appendix D, Figure D1. Only a weak correlation between *D*_L,COc,5s_ and Cobb angle was found (*r* = −0.29; *P* = 0.04).

**TABLE 3:** Comparison of dual test gas pulmonary diffusing capacity *Z*‐scores based on height versus arm span.

Variable	*Z*‐score on height	*Z*‐score on arm span	*P*
*D*_L,COc,5s_, *Z*‐score	−0.85 (1.02)	−1.05 (1.07)	0.33
*D*_L,NO,5s_, *Z*‐score	−0.62 (1.30)	−0.82 (1.32)	0.71
*D*_M,CO_, *Z*‐score	0.012 [−1.21, 0.63]	−0.23 [−1.31, 0.66]	0.58
*V*_C_, *Z*‐score	−1.02 [−1.82, −0.30]	−1.07 [−2.11, 0.41]	0.43

*Note*: Values are presented as mean (SD) or median [IQR]. Abbreviations: *D*_L,COc,5s_, pulmonary diffusing capacity for carbon monoxide corrected for haemoglobin measured with 5 s breath‐hold; *D*_L,NO,5s_, pulmonary diffusing capacity for nitric oxide with 5 s breath‐hold; *D*_M,CO_, membrane diffusing capacity; *V*_C_, pulmonary capillary blood volume.

## 4 DISCUSSION

In the present study, we found that about 39% of patients with idiopathic scoliosis exhibit either a reduced *D*_L, COc, 5s_ or a reduced *D*_L, NO, 5s_ 40 years after initial diagnosis with varying contributions from *V*_C_ and *D*_M, CO_. Moreover, no significant pattern between smoking status and reduced diffusing capacity metrics was found. Lastly, no apparent relationship between scoliosis severity and pulmonary diffusing capacity metrics was detected, and the findings were largely similar when based on height contra arm span data. To our knowledge, no previous studies have determined *D*_L, CO, NO_ metrics in patients with idiopathic scoliosis 40 years after diagnosis. When compared to the more widely used standard single‐breath technique for *D*_L, CO, 10s_, *D*_L, CO, NO_ using a 5 s breath‐hold time instead for measurement of CO and NO transfer simultaneously provides a view of the factors influencing pulmonary gas exchange. However, it should be noted that *D*_L, CO, 5s_ is known to be slightly albeit consistently lower than *D*_L, CO, 10s_, likely due to differences in inhaled gas composition and effects of NO on CO uptake kinetics (Nymand et al., 2024; Ogilvie et al., 1956; Thomas et al., 2014). In principle, NO may be considered a ‘purer’ measure of pulmonary diffusing capacity than CO, because NO reacts with haemoglobin approximately 1500 times faster than CO (Zavorsky & van der Lee, 2017). Indeed, it is these differences in uptake kinetics between NO and CO that permit the mathematical derivation of *D*_M, CO_ and *V*_C_ (Krogh, 1914; Zavorsky & van der Lee, 2017). Thus, the finding that patients with reduction in *D*_L, COc, 5s_ mainly showed reductions in the *V*_C_ component suggests pulmonary microvascular impairment; however, varying contributions from *D*_M, CO_ and *V*_C_ were found.

It has been suggested that if scoliosis appears before full lung development, this might impair alveolar development contributing to hypoplasia of alveoli and pulmonary vasculature causing reduced alveolar volume and reduced pulmonary vascular beds (Davies & Reid, 1971). Reduced microvascular growth might lead to diminished *V*_C_, thus resulting in decreased diffusing capacity. This might also lead to a reduction in *D*_M, CO_ from reduced alveolar–capillary membrane surface area. Furthermore, curvature of the spine could result in lung compression causing reduced lung growth, possibly interfering with microvascular growth, and failure of alveolar enlargement. Extrapulmonary restrictions including obesity, respiratory muscle insufficiency and kyphoscoliosis found in scoliosis patients might also contribute to this (Ragborg et al., 2024). Additionally, the use of bracing as treatment has also been suggested to cause limited lung growth, thus also contributing to impaired alveolar expansion (Kennedy et al., 1987). This suggests that thoracic cage deformity may contribute to impaired diffusing capacity, as this might impair both alveolar and microvascular growth resulting in reduced alveolar–capillary membrane surface area (Abdelaal et al., 2018; Karol et al., 2008; Koumbourlis, 2006; Ruiz‐Juretschke et al., 2017; Takahashi et al., 2006). However, if this were the only mechanism, the degree of pulmonary diffusing capacity impairment would be expected to correlate with scoliosis severity, which was not observed with the exception of a weak negative correlation between scoliosis severity and *Z*‐score for *D*_L, COc, 5s_ when using arm span data. This is in line with a previous study by Takahashi et al. (2006) finding no correlation between spine curvature and reduced diffusing capacity (Takahashi et al., 2006). This contrast with other previous studies that have suggested that a big Cobb angle and thus severe scoliosis disease might affect pulmonary diffusing capacity across different severity thresholds (Byun et al., 2020; Kan et al., 2023; Mbamalu et al., 2023; Nachemson, 1968; Tsiligiannis & Grivas, 2012). This discrepancy may reflect that our cohort presented with Cobb angles ranging from 20° to 65°, which may not be severe enough for detecting stronger associations, with earlier studies suggesting Cobb angles, of more than 100° to cause pulmonary impairments (Weinstein et al., 1981). In any event, further studies are warranted to determine the exact cause of the observed reduction in both *D*_M, CO_ and especially *V*_C_ across the scoliosis severity spectrum. In addition, given that a substantial proportion of the current population were former or current smokers, one may think this could to some extent explain these findings. Conversely, we found no pattern between having reduced pulmonary diffusing capacity metrics and smoking history.

Height and arm span are important determinants in predicted pulmonary function and thus play an important role in assessing the degree of pulmonary function impairment, with arm span being used as a proxy for height when it is not possible to obtain a valid height measurement (Chabra, 2008). Accordingly, height, but not arm span, differed between the scoliosis severity groups. Thus, spinal deformities depending on the severity can lead to loss of height, which causes discrepancies upon calculation of predicted values and thus differences in the calculation of *Z*‐scores and determination of pulmonary function impairment. More severe spinal deformity might therefore cause higher discrepancy in *Z*‐scores for pulmonary function causing underestimation of pulmonary diffusing capacity metrics. In a previous study, significant differences in *Z*‐scores for FVC and TLC calculated upon the use of height and arm span were found, illustrating patients with reduced FVC and TLC when measurements conducted upon height and arm span were compared (Ragborg et al., 2024). This suggests a possible reduction in lung size compared to the predicted lung size due to disease illustrated when using arm span‐derived data. However, in our study we detected no significant difference in *Z*‐scores between height and arm span regardless of disease severity for the 51 patients where arm span measurements were available. This is also supported by the study by (Ragborg et al., 2024) finding acceptable agreement between measurements conducted on height and arm span for pulmonary function metrics (Ragborg et al., 2024). Even so, in this study we found that if anything, height slightly overestimated *D*_L,COc,5s_ and *V*_C_, whereas no overall difference was found for *D*_L,NO,5s_ and *D*_M,CO_. Our data suggest that arm span and height could possibly be used interchangeably in clinical settings, as no overall significant differences were found according to *Z*‐scores. This could be due to less severe spinal deformity in patients of this study, and the assessment of differences in arm span and height‐derived data still needs to be conducted in more severe disease stages.

From a clinical perspective, the relationship between the degree of dyspnoea and reduced *D*_L,CO,NO_ in treated idiopathic scoliosis patients remains unclear and has not been thoroughly investigated. Our findings demonstrate no consistent correlation between scoliosis severity and *D*_L,CO,NO_, suggesting that dyspnoea may not be directly linked to the degree of spinal deformity. Studies on conditions such as COPD patients, smokers with mild airway obstruction and healthy individuals following COVID‐19 infection have shown a strong correlation between reduced pulmonary diffusing capacity and persistent dyspnoea (Behnia et al., 2017; Elbehairy et al., 2017). It is not yet established whether a similar relationship exists in patients treated for idiopathic scoliosis. However, as reduced *D*_L,CO,NO_ impairs gas exchange, it could potentially contribute to dyspnoea regardless of the underlying reason for the reduced *D*_L,CO,NO_, but this study was not designed to investigate whether reduced *D*_L,CO,NO_ is a driver of dyspnoea in scoliosis patients.

There are some limitations to this study that must be considered. As both *D*_M,CO_ and *V*_C_ are determined from the measurements of *D*_L,CO,NO_, there is an involvement of several assumptions and empirical constants that might challenge the use of these variables (Borland & Hughes, 2019). The diffusivity ratio α is assumed to be 1.97, whereas other studies have suggested greater α values trying to account for discrepancies between methods. However, this can mostly be dismissed as they differ from the physical diffusivity ratio resulting in inconsistent α values and until more research is carried out on NO and CO tissue diffusivities in the lungs, an agreement of an α value on 1.97 persists (Zavorsky et al., 2017). Additionally, in this study θ_NO_ is assumed a finite value, whereas other studies suggest for an infinite value of θ_NO_ due to the fast reaction rate of NO with Hb. However, a resistance to combination of NO and the red blood cell exists and θ_NO_ cannot be infinite, which aligns both with theoretical predictions and in vitro and in vivo experimentation, and thus a consensus exists for a finite value for θ_NO_ (Zavorsky et al., 2017). Also, θ_CO_ is based on empirical constants obtained at pH 7.4, rejecting previous values based on non‐physiological pH measurements. To determine a possible reduction in the pulmonary function metric, the use of *Z*‐scores has been included. Using *Z*‐scores gives a better understanding of possibly diminished pulmonary function by taking measured values, predicted value and between‐subject variability into consideration. Using *Z*‐scores applies the same cut‐off to all sex, ages, ethnicities and metrics, thus making the use of *Z*‐scores more consistent across different ages and sex giving an idea about how far the measured pulmonary function metric is placed from the predicted value.

Even though idiopathic scoliosis is a relatively uncommon disease, our small study size could inflict type 2 errors to some degree. Furthermore, as idiopathic scoliosis is more common in women (Sung et al., 2021), we only had one male included in the study. Another limitation is the lack of a control group for comparison of the results to the healthy population, which only gives the study an insight into the pulmonary diffusing capacity 40 years after diagnosis. Lastly, measurements of *D*_L,COc,5s_, *D*_L,NO,5s_, *V*_C_ and *D*_M,CO_ have not been conducted at the time of diagnosis and a potential alteration to these metrics from diagnosis to 40 years after diagnosis cannot be determined. Nonetheless, this is one of the first studies to assess diffusing capacity by *D*_L,CO,NO_ 40 years after scoliosis diagnosis, and treatment has been completed in a representative cohort of idiopathic scoliosis patients ranging from mild to severe disease at the time of measurement.

### 4.1 Conclusion

Forty year after the initial diagnosis of JIS or AIS, 39% of patients exhibited either a reduction in *D*_L, COc, 5s_ or a reduction in *D*_L, NO, 5s_ across all scoliosis severities with varying contributions from *V*_C_ or *D*_M, CO_, but with no correlation to scoliosis severity. Furthermore, using arm span data as a substitute for height did not affect any of the *Z*‐scores for *D*_L, CO, NO_ metrics, indicating that height and arm span can be used interchangeably for appropriate classification of dual test gas pulmonary diffusing capacity impairment in this population.

## AUTHOR CONTRIBUTIONS

Data collection: Rie S. Thomsen, Milan Mohammad, Lærke C. Ragborg and Jann Mortensen. Data analysis: Rie S. Thomsen, Milan Mohammad and Jann Mortensen. Data interpretation: Rie S. Thomsen, Milan Mohammad, Lærke C. Ragborg, Casper Dragsted, Søren Ohrt‐Nissen, Martin Gehrchen, Benny Dahl, Ronan M. G. Berg and Jann Mortensen. Preparation of figures: Rie S. Thomsen and Milan Mohammad. First draft: Rie S. Thomsen, Milan Mohammad and Ronan M. G. Berg. Revisions: Rie S. Thomsen, Milan Mohammad, Lærke C. Ragborg, Casper Dragsted, Søren Ohrt‐Nissen, Martin Gehrchen, Benny Dahl and Jann Mortensen. Supervision: Ronan M. G. Berg and Jann Mortensen. All authors approved the final version of the manuscript and agreed to be accountable for all aspects of the work in ensuring that questions related to the accuracy or integrity of any part of the work are appropriately investigated and resolved. Jann Mortensen is the guarantor of this work and accepts full responsibility for the work and the conduct of the study, has access to the data and controls the decision to publish. All persons designated as authors qualify for authorship, and all those who qualify for authorship are listed.

## CONFLICT OF INTEREST

None declared.

## Data Availability

The data underlying our findings can be shared upon reasonable request and directed to the corresponding author.

## APPENDIX A

### A.1

**TABLE A1:** Dual test gas pulmonary diffusing capacity *Z*‐scores based on height.

	Total (*n* = 57)	Mild (*n* = 13)	Moderate (*n* = 22)	Severe (*n* = 22)	*P*
*D*_L,COc,5s,_ *Z*‐score	−0.92 (1.07)	−0.71 (1.05)	−0.96 (1.13)	−1.02 (1.05)	0.70
*D*_L,NO,5s_, *Z*‐score	−0.72 (1.30)	−0.61 (1.27)	−0.85 (1.16)	−0.65 (1.49)	0.83
*D*_M,CO_, *Z*‐score	−0.14 [−1.28, 0.59]	−0.35 [−0.99, 0.52]	−0.36 (1.35)	0.40 (2.47)	0.71
*V*_C_, *Z*‐score	−1.04 [−1.85, −0.36]	−0.90 (0.95)	−0.75 (1.82)	−1.42 [−1.95, −0.72]	0.50
*K*_COc_, *Z*‐score	−0.05 (1.89)	0.55 (2.36)	0.05 (1.72)	−0.49 (1.72)	0.28
*K*_NO_, *Z*‐score	0.27 (2.27)	0.80 (2.54)	0.27 (1.97)	−0.05 (2.42)	0.58
*V*_A,5s_, *Z*‐score	−0.94 (1.32)	−1.24 (1.55)	−1.09 (1.22)	−0.61 (1.25)	0.31

*Note*: Values are presented as mean (SD) or median [IQR]. Abbreviations: *D*_L,COc,5s_, pulmonary diffusing capacity for carbon monoxide corrected for haemoglobin measured with 5 s breath‐hold; *D*_L,NO,5s_, pulmonary diffusing capacity for nitric oxide with 5 s breath‐hold; *D*_M,CO_, membrane diffusing capacity; *K*_COc,_ rate of change of CO from alveolar gas corrected for haemoglobin; *K*_NO_, rate of change of NO from alveolar gas; *V*_A,5s_, alveolar volume at 5 s breath‐hold; *V*_C_, pulmonary capillary blood volume.

## APPENDIX C

### C.1

**TABLE C1:** Influence of smoking, treatment and age of diagnosis on pulmonary diffusing capacity metrics.

Variable	*D*_L,NO,5s_	*D*_L,COc,5s_	*D*_M,CO_	*V*_C_
Age of diagnosis	0.17	0.07	0.21	0.52
Smoking (yes/no)	0.18	0.19	0.49	0.79
Treatment (yes/no)	0.43	0.50	0.36	0.78

Data are represented as *P*‐values for the given correlation. Abbreviations: D_L,COc,5s_, pulmonary diffusing capacity for carbon monoxide corrected for haemoglobin measured with 5 s breath‐hold; *D*_L,NO,5s_, pulmonary diffusing capacity for nitric oxide with 5 s breath‐hold; *D*_M,CO_, membrane diffusing capacity; *V*_C_, pulmonary capillary blood volume
